# Reel worlds: Reconstructing the history of postwar child psychotherapy through fiction film

**DOI:** 10.1177/0957154X261430318

**Published:** 2026-03-31

**Authors:** Tim Snelson

**Affiliations:** 1 University of East Anglia, Norwich, Norfolk, UK

**Keywords:** film, cinema, psychotherapy, child psychology, Margaret Lowenfeld, Institute of Child Psychology

## Abstract

This article uses British drama film *No Place for Jennifer* (1950) to argue for the evidential value of staged content of real psychotherapeutic places (Institute of Child Psychology) and practices (Margaret Lowenfeld’s World Technique). In the absence of ‘real’ documentary footage, discoveries of recreated therapeutic spaces within largely forgotten genre films can help reconstruct histories of the ‘psy’ sciences whilst offering insight into the contestations specific sites and practices provoked. Employing Baron’s reformulation from ‘documentary’ to ‘archival document’, it demonstrates that content extracted from previously disregarded films can fill archival lacunas whilst expanding both ‘what can be said’ about the past and how it can be said.

## Introduction

The last decade has seen a growing historical inquiry into audiovisual material produced as part of or as objective record of psychiatric research. The resultant film content is typically evaluated for its value as an indexical record of the medical practices and/or spaces that it depicts. But what about dramatised films, even commercial feature films produced primarily as entertainment? Do they possess epistemic value as historical evidence? Whilst caution must be taken to neither view nor present such films as factual, media scholars would extend the same concern to other forms of mediation, including ‘documentary’ footage shot by scientists themselves within laboratory or medical settings. All media is mediated, its content is selected, edited and framed in a purposeful way to make a particular point. Whilst the article will mention a number of postwar British films, the main case study will be the British melodrama *No Place for Jennifer* (1950) which addresses the psychological effects of divorce on a young girl. The film features a character based on child psychotherapist Margaret Lowenfeld and uses her World Technique (the original sand play therapy) as a key plot device. The film is an adaption of a novel written with input from and observation within Lowenfeld’s Institute of Child Psychology (ICP), a largely undocumented space that was authentically recreated as a studio set for the film. In using the film to explore and understand the interrelated spatial coordinates and social dynamics of Lowenfeld’s clinic, this article is in dialogue with previous research in this journal. This includes McGeachan’s (2014) work on the vital role of ‘spatial thinking’ in R. D. Laing and Aaron Esterson creation of radical psychotherapy clinics, and Pérez-Fernández and López-Muñoz’s (2019) research on the ongoing influence of architect Thomas Kirkbride’s design for nineteenth century mental institutions on Hollywood’s dominant representational trope of the ‘asylum of terror’.

Following Joice’s (2022) work on the epistemological value of archive films to the history of psychiatry, I will analyse *No Place for Jennifer* in relation to her triumvirate of evaluative issues – evidence, ethics and empathy – to explore whether staged archival records can help us understand and resituate real science within its converging historical contexts. This article argues that the film is historically significant because, as far as we know, there is no film footage of the real ICP or Lowenfeld doing sand tray therapy. There are some photos of her kneeling by a sand tray and of children’s completed ‘Worlds’, but no surviving footage of the dynamic therapeutic process as detailed in *Jennifer*. Cinematically, the use of Lowenfeld (rather than Freudian psychoanalysis) is important for power dynamics between doctor and patient, placing the child at the centre with the psychotherapist observing. In these respects, the film is also significant in terms of the issues of ethics and empathy, filling a specific archival lacuna whilst circumventing (or at least reducing) some of the concerns of factual footage regarding doctor-patient power relations and their recreation within the production process and onscreen.

## From documentary evidence to ‘archival documents’

Over the last decade, a global field of study has developed to explore the epistemic value and ethical implications of audiovisual archives relating to the history of the ‘psy’ sciences (psychiatry, psychoanalysis, psychology, and related disciplines). This research emerges from, and is at the intersection of, recent interest within film studies in types of non-commercial ‘useful film’ (Acland and Wasson, 2011) previously disregarded by the discipline, as well as a greater openness within historical scientific inquiry (in part prompted by the interdisciplinary medical humanities project) to take audiovisual materials more seriously as valuable sources within their own right (Berton and Köhne, 2027; Curtis, 2016; Killen, 2017; Winter, 2004).

Some of the most interesting and enlivening work in this field focuses on child psychology and psychotherapy, thinking beyond issues of documentation or circulation to explore the concrete role of film in the development of theories and practices within the mind sciences. Within the British context, notable recent work includes Joice’s (2022, 2023) research on the use of cinematic microanalysis in the study of mother-child attachments and Evans and Harbord (2024) scholarship, both individually and collaboratively, on the vital role of film in early child development studies (Evans, 2024a, 2024b) and in identifying autism as a discrete condition in the 1950s (Harbord, 2024, 2025). This research focuses on factual film content, produced by or at the direction of psychiatric professionals, and intended to be used ‘diagnostically and curatively, as documentary proof, teaching aid, provocation and exposé’ (Joice, 2022: 4).

What is excluded from this list is entertainment. Understandably, perhaps, this type of serious scientific filmmaking is perceived to be diametrically opposed to the entertainment function of mainstream filmmaking. Research on commercial, popular films that focus on psychiatric subjects typically prioritise questions of accuracy and affect – in the worst cases, their dangerous spread of disinformation about medical conditions and professional practices (Leistedt and Linkowski, 2013; Swart, 2016). Science communication research has been more open to the potential value of (some) such material to circulating scientific ideas or debunking unhelpful myths (Gouyon, 2015; Kirby, 2013). But a distinction persists in placing fiction films’ value (or problem) in its persuasion of a lay public, and documentary films’ potential to authentically capture or even create science for an educated elite. Maybe fiction film can offer something more to scientists and historians, whilst more caution is required in the evidentiary use of factual medical films?

Recent work on archives and documentary seeks not only to acknowledge, but also embrace and exploit, the ‘unruliness’ of the archive and de-hierarchise types of ‘archival documents’. Baron’s (book *The Archive Effect* is useful in calling for a ‘reformulation of “the archival document” as an *experience of reception* rather than an indication of official sanction or storage location’ (p. 7), as an ‘“archive effect” [that] allows us to account for variable experiences of reception of the same text [. . .] across the generic boundaries of films usually categorized as documentary, experimental, fake documentary, and even fiction film’ (p. 10).

This has been the principle underlying my own archival research into creative collaborations between filmmakers and mental health professionals in the long 1960s (Snelson et al., 2024a) and the creation of new, as Baron refers to them, ‘appropriation films’ for display on gallery at the Science Museum that use clips from feature films to add historicity to the psychiatric objects on display (Snelson et al., 2024b). This article goes a step further in assessing the value of staged filmic records of real medical places and practices, not just as examples of creative coproduction or public engagement tools, but as research documents in their own right.

Within her field of political communication history, Herbst (2001) has similarly employed popular films for such archaeological purposes as ‘public opinion artifacts’ (p. 465). She demonstrates how Hollywood film *Mr. Smith Goes to Washington* (1939) offers historical evidence of the ‘public opinion infrastructure’ in the 1930s that not only complements but also, in key respects, counters dominant historical accounts relying heavily on data drawn from Gallup and other organisations within the ‘burgeoning survey industry’ who benefited from the idea of an informed and rational public (p. 462). Through this intervention in her own field, Herbst also contributes to film criticism by moving beyond authorial intent – how the film’s confused political message relates to director Frank Capra’s complicated pollical beliefs and the broader political climate – to focusing on how the film might provide historical evidence of public opinion and, indeed, ‘how the public might have seen itself’ (p. 462).

*No Place for Jennifer* might be considered similarly as a valuable ‘artifact’ for investigating, in this case, the converging ideas, institutions and infrastructures of child psychotherapy. In film studies, *No Place for Jennifer* has largely been discussed in the context of a cycle of post-war British films about the family, attachment and divorce that seek to ‘reconstitute’ the family unit following the disruption of World War Two (Fink, 2013; Geraghty, 2000; Gillet, 2017). In these chapters, *Jennifer* is discussed in a broader context of psychological and psychoanalytic research on wartime separation and attachment theory – namechecking Anna Freud, John Bowlby and, to a lesser extent, Donald Winnicott – but makes no mention of Lowenfeld or her Children’s Clinic which, as discussed below, differed in key respects from these other approaches. Whilst this research will, like Herbst’s, compliment this film criticism by offering a more focused perspective on the film, the purpose of this article is to use *Jennifer* to demonstrate the historical value of fiction film as archival evidence of clinical practices and places whose understanding would benefit from audio-visual record.

My central case study is Margaret Lowenfeld’s Institute of Child Psychology in London, as depicted in *Jennifer*, and argues that staged records such as those depicted in the film can serve to fill archival lacunas when no existing or accessible archive film of such important spaces of psychotherapeutic practice exists. Secondly, it will highlight how such records address some of the ethical issues in relation to depictions of real mental health service users and informed consent. I am mindful, however, that such depictions, employing actors or, perhaps, consenting non-actors with lived experience, raise other ethical issues as these films draw upon real case history material. Finally, I will consider such fictive depictions in relation to debates about the elision of patient experience and doctor-patient power relations (often discussed through the idea of the ‘medical gaze’ (Foucault, 1973 [1963])), given these films centre patients as their lead protagonists and purport to be privileging their perspectives.^1^ The article combines historically-contextualised close textual analysis of *No Place for Jennifer* and related archival documents (including the shooting script, studio publicity material, promotional images, correspondence) with a critical reception studies approach, evaluating these ‘archival documents’ as a mode of address, circulation and reception.^2^

## Lowenfeld and the Institute of Child Psychology

Margaret Lowenfeld was a vital figure in child psychology and paediatric medicine, who was marginalised both professionally in her lifetime and in subsequent histories of the field. In part this was as she went against the psychoanalytic norms of the profession and, later, resisted incorporation and adaptation of her practise for the NHS (Urwin, 1988: 107), perhaps also because she didn’t align with traditional gender (and sexual) expectations within her life and work (Hubbard, 2025). Lowenfeld’s disruption of professional (perhaps also social) convention was partly responsible for the marginalisation of her work, both in her time and in subsequent histories of childhood and the ‘psy’ sciences. Sigmund Freud was amongst Lowenfeld’s eclectic influences, but she developed a growing antipathy towards psychoanalysis; she had an antagonistic relationship with Melanie Klein (who was dismissive of Lowenfeld’s approach) but also refused to align herself with opposing factions (including the Anna Freudians) within or outside of the ‘controversial discussions’ (King and Steiner, 1991). Whilst there were similarities between the psychodynamic underpinnings of Lowenfeld and psychoanalytic approaches of contemporaries Klein and Anna Freud, Lowenfeld was interested in the transference between child and object rather than between child and therapist (Lowenfeld, 1955). She consciously sought to detach child psychotherapy from the limitations of the ‘talking cure’ by looking beyond the rigidity of her field and its established canon.

In the 1980s, psychologist Cathy Urwin sought to redress the elision of Lowenfeld’s life and work with her comprehensive *Child Psychology, War and the Normal Child*. The book combined a detailed biographical account of her career with selected papers authored by Lowenfeld that foreground her key theoretical ideas. Whilst Lowenfeld’s practice has lived on (largely uncredited) across a range of global sand tray and play therapy practices, her theory is largely forgotten (Hutton, 2004). In recent years, historians of childhood and the ‘psy’ sciences have sought to reconnect her theory and practice in a series of articles that seek to foreground the eclectic and interdisciplinary nature of her work and its resultant influences across a range of scientific, educational and artistic fields (Hayward, 2019; Hubbard, 2025; Joice, 2026; Snelson et al., 2024b). A key interest for the above scholars is in relation to Lowenfeld’s at times controversial blurring of the boundaries of the mental health profession with other fields – education, anthropology, media, even spiritualism – that she felt could help in understanding children and improving their lives.

Lowenfeld looked to influential contemporary developmental and educational psychologists, particularly Jean Piaget and Cyril Burt, but also to philosophy (particularly through her productive intellectual relationship with British historian and philosopher R. G. Collingwood), modernist art, fairy tales and folklore, and contemporary popular culture. Her book *Play in Childhood* (1935) draws upon these diverse influences to explain and analyse her practice and research with hundreds of children within the ICP. Seeing play as central to children’s early cognition, development, social learning and emotional attunement, the book offers a nuanced theory of ‘free play’ that recognised both its functional role in helping children move through developmental stages, but also its vital expressive role in bringing their pre-verbal experiences into the present and making them legible (primarily to themselves rather than to the therapist).

In 1928, Lowenfeld invented the World Technique (WT) to instrumentalise this expressive function of play and make it visible to both child and therapist, taking key inspiration for the originary form of sand tray therapy from H. G. Wells’ popular books *Floor Games* (1911) and *Little Wars* (1913). The WT – a child-led psychotherapeutic treatment rather than diagnostic or projective tool – required a metal tray (75 × 50 × 7 cm), wet and dry sand, and a vast cabinet of toys: miniature figures (varied people, domestic and wild animals, fantasy figures); model buildings and scenery; transport (cars, trains, boats); plasticine. The toys employed in the technique were not specially made for this purpose, but mass produced by brands like Britains and Meccano from the early 20th century onwards (Hayward, 2019). The scientific instruments required were not propriety or protected – like the Rorschach inkblot tests – but purposefully easily available and added to, with Lowenfeld encouraging practitioners to bring in toys relevant to their national cultures and localities. Through the WT, Lowenfeld turned commercially produced toys into therapeutic tools and flipped power relations between therapist and patient. The material nature of Lowenfeld’s approaches – the centrality of physical objects and media – has been an obstacle to the academic understanding and recognition of her work. Archives and museums have been more receptive to Lowenfeld’s importance than academia with, for example, the Science Museum’s Wellcome medical galleries foregrounding the importance and visual nature of her work by displaying a selection of her toys and recreated Worlds in a permanent *In the Therapist’s Room* display.

The WT was practiced at Lowenfeld’s ‘Children’s Clinic’ in London alongside other play therapy techniques including her other major invention, the Lowenfeld Mosaics (invented 1948). She set up the ‘Children’s Clinic for the Treatment and Study of Nervous and Difficult Children’ in 1928, which moved to larger premises and then became the ‘Institute of Child Psychology’ (ICP) in 1931. The location moved around West London for a decade but established at 6 Pembridge Villas, W.11 until 1979 when the Clinic closed following Lowenfeld’s death. The Clinic included various playrooms including the ‘water room’ and ‘mess room’ and playroom for sand tray therapy (that also included an oversized doll’s house that children could climb into).^3^ This lengthy quote from *World Medicine* (1969) gives an indication of the architectural layout of the building and also, importantly, the power relations therein:

In the six rooms and enclosed garden which form the children’s sanctum at the Institute of Child Psychology in Bayswater, London, almost anything goes. There are no rules or restrictions for the children, except [a few. . .] to prevent a particularly exuberant child damaging himself or another child or wrecking the furniture. For the adults observing the children at work one paramount rule is their guide: the standards of behaviour in the playrooms are the standards of children and not of adults [. . .] From the beginning each child is told that whatever he does in the playroom has a meaning: no matter what form of activity he chooses—and the choice is the child’s—he is expressing some thought or emotion, and together the child and the therapist will try to work out the significance of his play.

The Clinic worked on the principle of child and therapist working things out together, at least in principle flattening doctor-patient hierarchies. The children (aged 4–18) were free to move between rooms and activities as they chose. The mostly female staff were anonymised in green overalls and instructed to be as least disruptive and interventionist as possible. No other adults, including parents, were allowed in the playrooms. This might in part explain why there is no film footage (and limited photographic evidence) of the playrooms within the Children’s Clinic.

However, the lack of film footage can equally be ascribed to Lowenfeld’s conflicted relationship with film and later television as mediums for research and communication. As we saw above, she appreciated the value of popular culture to her creation and children’s creative use of the WT. In a 1937 speech to the British Psychological Society (where Carl Jung first took an interest in her approach) she highlighted that the Worlds children created often drew on visual storytelling techniques aligned and inspired by popular film and film serials. She explained they ‘may be vivid and moving, suggesting a cinema film more than anything else for the rapidity of movement. The ‘World’ of many weeks may show a continuous story, each successive afternoon’s work being the development and continuation of the previous one’ (Lowenfeld in Urwin, 1988: 247). Lowenfeld’s position on the significant influence of cinema on children was conflicted but realist. Whilst she shared broader political and social concerns about children watching ‘unsuitable content’ she resisted joining calls to ban popular children’s cinema clubs. When called upon to comment on the issues she responded, ‘There is no way of stopping them. The thing do is work with them’ (*Daily Mirror*, 1931: 3).^4^

Whilst she admired the ethnographic use of film by colleagues such as Margaret Mead, Lowenfeld was reluctant to use film in her own research or dissemination of her theory and practice, instead employing grids, sketches, illustrations by commissioned artists and ‘aerial photographs’ of the Worlds. In the late 1950s, she started to consider using film as a ‘a record of the whole process of World-making as seen from inside by the maker’ (Lowenfeld quoted in Joice, 2026: 22), and in 1965 began a Worlds film project with the Royal College of Art (with Mead as advisor) but no footage of the experiment survives (Joice, 2026). We do, however, have the reconstructed record of the ICP and the WT within *Jennifer*.

## *No Place for Jennifer* and the ‘children’s clinic’

*No Place for Jennifer* is a 1950 British melodrama directed by Henry Cass for the Associated British Picture Corporation. In the film, 9-year-old Jennifer (Janette Scott) is devastated when her divorced parents – her father William (Leo Genn) and mother Paula (Beatrice Campbell) – announce their intentions to marry new partners. Already an anxious child, Jennifer becomes increasingly distressed as she experiences the emotional upheaval of being torn between two households. Her mounting anxiety leads to behavioural issues both at home and at school. Concerned about her well-being, the school persuades her father to send Jennifer to a special clinic for maladjusted children. Jennifer responds well to the treatment but her mother takes her out of the clinic and plans to take her to Paris. Her father intervenes and a court battle over custody ensues. This further traumatises Jennifer, who runs away from the court and goes missing. After some unsettling encounters on the streets of London, Jennifer is found and brought home. Her parents decide that Jennifer will not be happy with either of them and arrange for her to move in with the stable and loving Marshall family who live next door. The film concludes somewhat hopefully with Jennifer happy with her new living arrangements.

**Figure 1.**
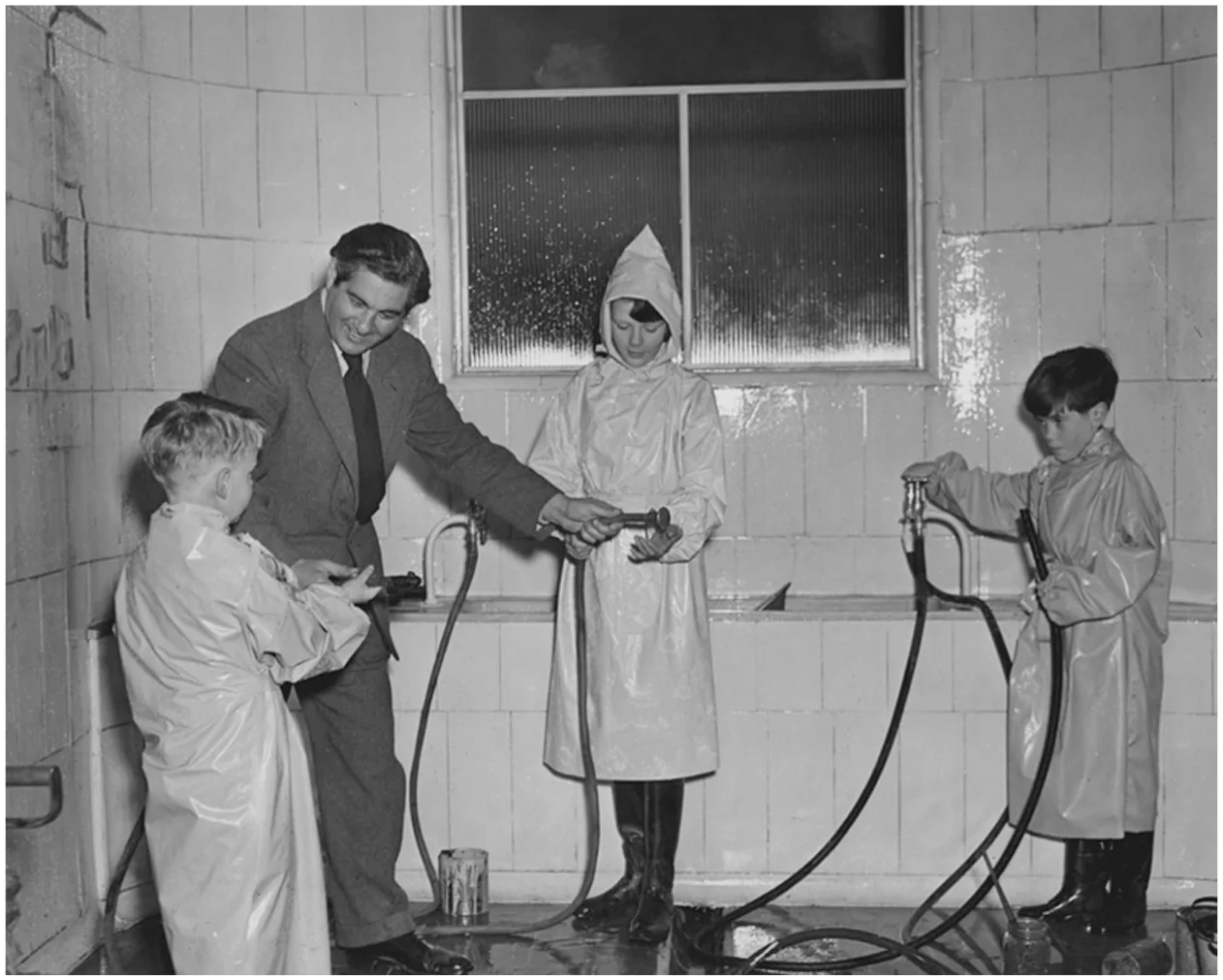
Henry Cass directs children in the ‘water room’. Source: Image courtesy of StudioCanal.

The production company Associated British was mostly recognised for routine rather than prestige productions, known by industry and audiences as makers of programmers and ‘B’ pictures based on established genres and existing properties. *Jennifer* was adapted from the 1948 middlebrow novel *No Difference to Me* by Hambledon (1948) and was identified as a ‘moderate “programmer”’ by the trade press (*Kinematograph Weekly*, 1950). The sections of the novel set in the ‘Children’s Clinic’ were written with input from staff in the ICP – an acknowledgment in the book thanks the ‘personnel of the Children’s Centre (Institute of Child Psychology) for their help in the writing of Chapter IX’). Lee-Thompson’s script follows chapter IX closely (though the therapeutic treatment is more complete in the film). *Jennifer* was not shot on location at the ICP but set designer Terence Verity meticulously recreated three of the playrooms as a ‘composite set’ on a sound stage in Welwyn Garden City as the film, publicity images and the script attest (see Figure 1).

The Children’s Clinic set features only in an 8 minute standalone section, that appears two thirds of the way into the film. When Jennifer’s dramatically changed behaviour in class (‘secretive, silent, nervy, definitely unhappy’) alerts her school to her inner turmoil, her father is encouraged to send her to the London-based ‘Institute of Child Psychology’, where she is treated by a character modelled on Lowenfeld, named simply ‘Doctor’. Whilst Jennifer’s father and step-mother are talking with the Doctor, another practitioner, Miss Gordon, shows Jennifer around the playrooms including the ‘Mess and Paint Room’ and ‘Water Room’ – both features of the real ICP – before introducing her to a third room where a number of children are engaging with the WT. Jennifer initially thinks the other rooms are ‘rather silly’ and favours the sand tray playroom, which allows her, with the help of the Doctor, to ‘get things straightened out together’. The ICP scenes compress time through the use of montage, dissolves and other editing techniques, between more lengthy dialogue-driven sequences. With the Doctor’s guidance, Jennifer works through the stages of chaos, struggle and resolution, expressed in the gradual ordering of her World and eventual removal of a symbolically-significant tiger figure (discussed in detail below), and at the end of the scene a hopeful Jennifer expresses a desire to keep coming to the Clinic. The Doctor stresses that Jennifer will ‘go on coming for a long time yet’, but the child’s estranged mother arrives unexpectedly to taker her ‘away from this horrible place’.

From an evidentiary perspective, the scene is valuable for understanding how Lowenfeld (1995 [1935]) worked within the ICP to facilitate autonomous play that was ‘spontaneous and self-generated’ (p. 23). Lowenfeld’s interest was in children’s individual expression rather than maternal or familial attachment. So, the therapist’s role was not to stand in for a parental figure, but to provide space for self-revelation through free play, as opportunities for such expressions had likely been stifled in the home environment. Also, whilst Lowenfeld, drawing upon Piaget, saw different types of play emerging at different stages (and structures *Play in Childhood*’s accordingly with chapters moving from infant’s individual and bodily play to the more social and interactive play of older children), she stressed that children might use all forms of play at any stage for different purposes. As we see within the scene, Jennifer is encouraged to move freely between the different playrooms and ‘regress’ to earlier modes of messy and wet play used by young children in ‘making intelligible to themselves the physiological processes they experience’ (Lowenfeld, 1995 [1935]: 83). The montage sequences and accompanying upbeat score, encourages us to see Jennifer’s subsequent joyful embrace of the ‘rather silly’ messy play as a breakthrough in her therapeutic journey.

In terms of the importance of material expression, the film shows Jennifer making a series of Worlds utilising many of the same toys and model buildings again. In this respect, the scene is useful in capturing the dynamic and multidimensional process of World building. The shooting script details Jennifer’s first experience of entering the playrooms, explaining, ‘The other children only give a cursory glance at Jennifer, and go on with their modelling and games. They pass a cabinet of trays that has practically every kind of model in it you can think of. Jennifer, speechless, can only stare in wonder . . .’ The WT required children’s free choice from a cabinet offering a near infinite selection of toys and models but, as Lowenfeld (1979) explained, children often reused the same objects across a series of Worlds, ‘rarely moving outside them’ (p. 66). The selected objects become invested with meaning for the individual child and become a lexicon by which they can articulate their overlaying past and present experiences and emotions. We see this in Jennifer’s shifting use and placement of the tiger in relation to the place of safety of her neighbours’ family home and, perhaps more explicitly, in the little boy Jeremy (who lost his parents in a plane crash) who uses violently crashing planes in his World building. Highlighting the centrality of restaging and repetition to the therapeutic process, Lowenfeld explains, ‘The life of a child, by externally and interiorly, is one of action. Their own experiences and those of the constituents of the world around them are seen by children as stories: stories which can be endlessly repeated or varied’ (Lowenfeld, 1979: 14). Whilst it might appear obvious, the visual medium of film is able to capture the active and affective nature of the children’s emotional making in a way that static illustrations and images – even with Lowenfeld’s detailed recording – cannot.

**Figure 2.**
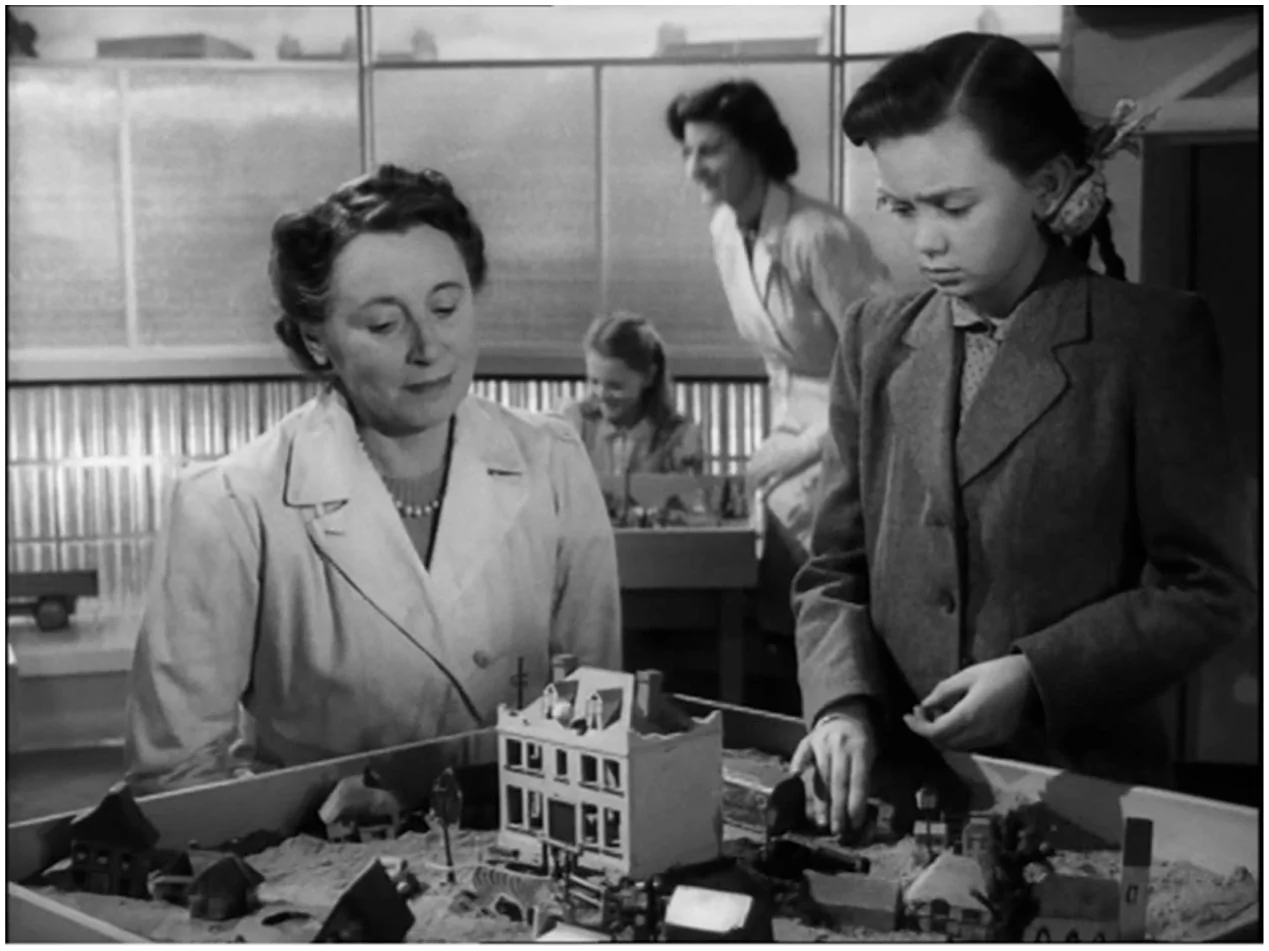
The doctor crouches into frame to view Jennifer’s first world. Source: Image courtesy of StudioCanal.

**Figure 3.**
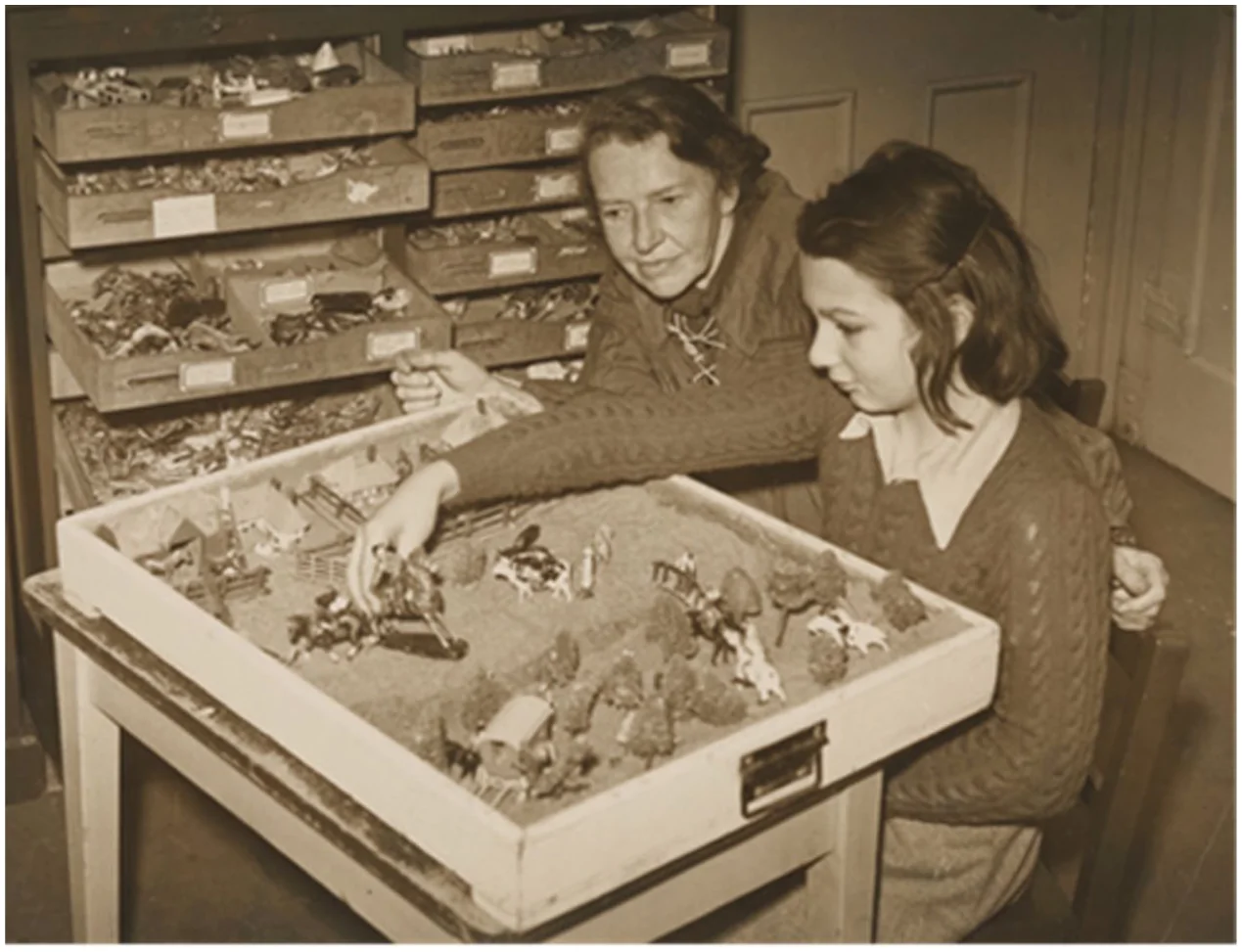
Margaret Lowenfeld observes a real child’s world. Source: Image courtesy of The Dr Margaret Lowenfeld Trust.

*Jennifer* is also useful in visualising the ICP’s principle of centring children’s perspectives and experiences and freeing them from the alien and anxiety-provoking world of adult discourse, including psychoanalytic thinking. The WT was a mechanism for joining children at their level and giving them a language to express their feelings in a way that felt favourable and familiar to them. At a formal level, this is expressed in the film through cinematography, through a number of point-of-view shots of the playrooms from Jennifer’s perspective and then, in the WT scenes, framing the image such that only the child and the sand tray are in frame, with the Doctor having to crouch down into frame to inquire about the meaning of the Worlds (see Figure 2). This mirrors the few existing images we have of Lowenfeld crouching down with a child (see Figure 3). The framing directs the Doctor’s ‘attention on an endeavour to discover what exactly the object used represents to the child who uses it’ (Lowenfeld, 1979: 7). This is reinforced in the dialogue exchange between Jennifer and the Doctor when she crouches into shot and, as the script explains, ‘studies her “World”’ (Lee-Thompson, 1949):

Doctor: ‘That’s a very tall house, isn’t it?’

Jennifer: ‘It was just like that’.

Doctor: ‘Oh, it’s a real house’.

Jennifer: ‘Yes, near where we used to live’.

Doctor: ‘Where was that?’

Jennifer: ‘Bringham, I used to have a lovely time there’.

Doctor: ‘I see there are a lot of wild animals prowling among the traffic. Now, I wonder what this tiger is doing here?’

Jennifer: ‘I don’t know really. But he belongs somehow. He’s dangerous’.

Throughout the sequence of Worlds, the Doctor character is seen asking what the objects mean to Jennifer rather than seeking to interpret them in the context of a standard set of interpretations.

Lowenfeld was clear that her approach was not about interpretation, but rather ‘careful inquiry’ to ‘discover what exactly the objects used represent to the child who uses them’ and then detailed recording’ of their use and the child’s shifting understanding of their meaning and function across a series of Worlds (p. 7). In this way it is very different from the work of her contemporaries, such as Anna Freud, who employed interpretation based on dream analysis and used toys primarily to enhance transference between therapist and child. Lowenfeld felt the therapist’s role was observer and recorder, as ‘memory’ rather than ‘reader’ of the Worlds. At the end of the sequence the Doctor asks, ‘Do you remember the first World you made?’ to which Jennifer responds, ‘It was a frightful muddle’. Lowenfeld saw children’s Worlds as the material residue of preverbal cognition and early bodily experiences; latent energies, including destructive ones, rather than unconscious desires or drives linked to infantile sexuality. Lowenfeld’s role was to facilitate creative ways to channel and communicate these energies through image making and messy play. At the start of the sequence when Jennifer is shown the mess room, in close up we see a small boy throwing buckets of paint at the wall and expressing intensely, ‘Every time I come here, I’m going to make a mess, mess, mess! Everything’s a mess! You’re a mess!’ before spitting on the ground. Miss Gordon looks pleased with the boy and comments to Jennifer ‘It’s rather fun, isn’t it?’ This simultaneous act of creation and destruction is understood as a ‘powerful release of vigour, of vitality’ (Lowenfeld, 1979: 25), a healthy expression of energies that had been previously blocked.

**Figure 4.**
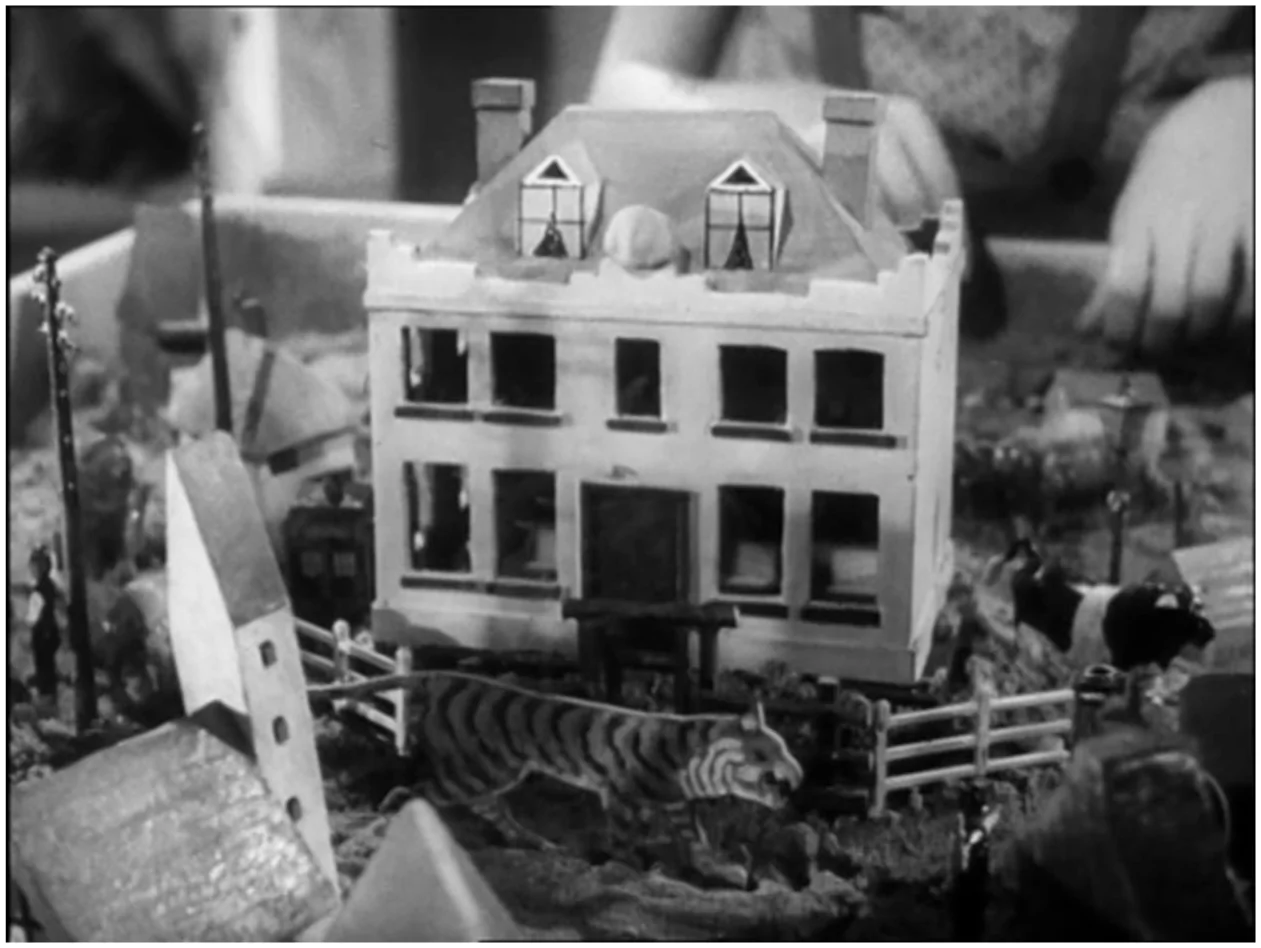
Jennifer’s ‘incomplete representational world’ jumbled by a tiger. Source: Image courtesy of StudioCanal.

Appropriate to cinema storytelling conventions, *Jennifer* depicts a treatment with a clear narrative logic, structuring the sequence around three of her Worlds that develop in character from ‘incomplete representational worlds’ (see Figure 4) – in which ‘real objects are put together in an unreal fashion’ – to ‘complete representational worlds’ (Lowenfeld, 1979: 7).^5^ Jennifer’s case history and presenting problems have clear parallels with one of the recorded treatments with Mary Smith (aged 10), detailed in *The World Technique*, whose trauma also manifested in behavioural problems at school. Lowenfeld explains, ‘Mary made use of objects representative of her ordinary surroundings, these were grouped in a way which expressed a constellation of ideas and emotions concerned with the family and her feeling towards and about her family’ (Lowenfeld, 1979: 62). The WT was about identifying and understanding ‘clusters’ of sights, sounds, memories, feelings bounded together in what the Doctor in the film describes as a ‘dangerous sort of a muddle’, that needed to be unmuddled and released. Jennifer’s ‘wild animal feelings’, as represented by the tiger, could easily be interpreted in Freudian terms of infantile sexuality but instead, in both Jennifer and Mary Smith’s cases, represent constellations of ideas and feelings about her toxic family environment that need to be articulated and expelled. Her complex feelings and fears about her parents have become ‘all mixed up’ with her anxieties about school, schoolmates’ gossip and about the divorce ‘being in the papers’. An earlier nightmare sequence uses a well-established superimposition technique to overlay images of Jennifer’s distressed sleeping face with alternating dissolves of fragmented objects and moments representing these tangles of emotions, experiences and relationships: a persistent ringing phone (representing her absent mother), cruel school-teachers and pupils, a violently ruined birthday cake, and so forth.

In the logic of the film (and Lowenfeld), Jennifer doesn’t need to work through unconscious Oedipal feelings about her mother and father as part of standard process of psychosexual development, but rather to get rid of her parents entirely as they are the source of her emotional problems (what is blocking or misdirecting her energies). Lowenfeld’s rationale of removing corrupting ‘adult systems of thought’ and the toxic effects of the dysfunctional parents on the child’s development, plays out in the film’s highly unusual ending in which Jennifer chooses to live with neither parent and instead moves in with the Marshalls next-door. This identification of and removal of the parent problem aligns with Lowenfeld’s ‘somewhat austere emphasis on early cognition and physical processes, at the expense of emotional attachments, [which] set her apart from her contemporaries in the field’ (Joice, 2026: 11). This proved a controversial aspect of both the film and Lowenfeld’s work.

## The historical reception of *No Place for Jennifer*

A historical reception studies approach employs discourse analysis of a wide range of paratextual materials (reviews, publicity materials, industry discourse) to seek to understand the ‘range of strategies available in social formations’ (Staiger, 1992: 80) that contemporary audiences might have brought to a film. In this way, it is alive to complexity and contention rather than looking for consensus and cohesion. The historical reception of *No Place for Jennifer* might, therefore, offer supplementary evidence to us as historians of the ‘psy’ sciences in showing how film critics and other moral and cultural entrepreneurs responded to the clinical practice on display within the film. For example, the ending of *No Place for Jennifer* confounded some film critics in its insistence that the child needed to be fully removed from her toxic parents to guarantee her psychological safety. As one critic explained ‘the withdrawal of *both parents* from Jennifer’s life, and the parking of her on a motherly stranger, is the worst “happy ending” ever conceived by man’ (Graham, 1950). Other reviews employed similarly dismissive language to bemoan the film’s outcome of ‘parking her for good on a third party’ (*Sheffield Chronicle*, 1950).

These critics sought to undermine the validity of the psychotherapeutic approach within the ‘ritzy clinic, where nurse talk entirely in textbook platitudes’ (*Kinematograph Weekly*, 1950: 22) through sarcasm and scorn at the perceived ‘coddling’ of the child patients. As *The Spectator* reviewer ridiculed, ‘I am no child psychologist but, but I wonder very much whether Jennifer’s unhappiness would have been erased by that merry bout of paint-slinging and water-splashing she indulged in at the clinic for maladjusted children, (though the workers there were appallingly understanding)’ (Graham, 1950). Another critic purposefully misrepresents her ‘diagnosis’ at the ‘Institute of Child Psychology where they explain that she has an obsession with a tiger’ (*Observer*, 1950). These film critics’ dismissal aligns with the conflicted media discourse around the real ICP, with a number of negative newspaper articles in the 1930s on Lowenfeld’s ‘Naughty Boys Paradise’ and ‘Do-As-You-Please School’ opposing the newly established ICP on ideological as much as psychological grounds (*Daily Herald*, 1938; *Daily Mirror*, 1931; *Liverpool Echo*, 1931; *The People*, 1938). The film comments on the challenging and controversial nature of the ICP’s work – and its resultant negative reception within sectors of the media – in the Doctor’s explanation to Jennifer’s father, ‘We don’t always get it right and we receive a lot of criticism’. From the 1930s onwards, Lowenfeld embraced this image of her as a disrupter or provocateur, acting as media commentator on a range of child and youth-related issues including the mental health benefits of violent ‘penny dreadful’ comics (*Daily Mirror*, 1939), parents’ responsibility in the ‘Teddy Boy’ problem (McLeave, 1954), and overly-feminine and restrictive girls’ fashion (*Daily Telegraph*, 1933). When Jennifer expresses her anxiety about her parents’ divorce being in the ‘horrible’ newspapers, the Doctor counters, ‘I’m often in the papers [. . .] You see the papers are useful for telling lots of people something you what them to know’.

Some of the negative critical reception of *No Place for Jennifer* seemed to have been related more to a broader sense of an over-emphasis on child psychology and divorce themes in British films at this time. Prior to release, the British censor (BBFC) dismissed the film as, ‘Yet another “child psychology” story, based on the overworked theme that quarrelling parents can cause a “maladjusted child”’ (Kitchener, 1949). Whilst British cinema largely ignored children’s perspectives prior to the late 1930s, the emotional trauma of the Second World War – and resultant psychological research on attachment by prominent figures like Bowlby – prompted filmmakers to engage seriously with child psychology (Fink, 2013). In the immediate postwar period, the British government sought to use documentary film as educational tools to comprehend children’s perspectives and educate parents in developmental ideas. These films were intended for both mainstream cinemas and as training films within professional settings.^6^ The Central Office of Information commissioned a ‘Child Psychology’ series of short films, most directed by women, instigated by Margaret Thomson’s *Children Learning by Experience* (1946), a verité-style observational study of children’s tactile relationships and learning through play (Easen, 2021). Whilst Jane Massy’s *Your Children’s Sleep* (1947) employed point-of-view cinematography and discontinuity editing to place the spectator in the position of the child as they encountered daytime stimuli that impacted their sleep and dreams. Some of these filmmakers subsequently crossed over into commercial feature films (e.g., Thomson’s science fiction film *Child’s Play* [1954]).

In the late 1940s, popular filmmakers sought to both benefit from and invest in this interest in children’s inner worlds. In 1950 alone, for example, in addition to *Jennifer*, child psychology themes and child psychologists appeared in a range of genre films including *The Magnet* (1950), *The Girl Who Couldn’t Quite* (1950) and *Tony Draws a Horse* (1950). The latter is an interesting companion piece to *Jennifer*, in building its comedic narrative around the conflicting parental approaches of Tony’s modern psychotherapist mother – who celebrates and nurtures his destructive creative energies – and his authoritarian surgeon father, who wishes to punish Tony after he draws a horse, complete with reproductive organs, on his office wall. The issue of appropriate genres for such material was a concern for critics at the time. For example, the critics that derided *Jennifer* typically sought to deter serious audiences by positioning the film as a ‘melodrama’ or ‘weepie’ with a ‘feminine angle’ (‘Verdict: Take your hankies, girls’) (Whitley, 1950). Others complained that the film suffered from ‘an overdose of applied psychology’ unbefitting to its populist storytelling mode or intended audience (Whitley, 1950) or derided its casting of ‘a lady psychiatrist (yes, that too!)’ specifically (*Sheffield Chronicle*, 1950). The critics’ problematic gendered politics of taste applied to female audiences was extended here to the casting of a female mental health professional.^7^ Conversely, however, some critics appreciated the film’s atypical scientific approach to its material, stating, ‘It’s so seldom that we see child psychology treated in a manner which is at once convincing and unmelodramatic’ (Collier, 1950).

This small reception study highlights how the evidentiary value of staged footage such as *Jennifer*’s Children’s Clinic scene – which, as demonstrated above, enlivens understanding of the dynamic, material practice of Lowenfeld’s World Technique – can be supplemented through an appreciation of the ‘variable experiences of the same text’ (Baron, 2013: 10) by a range of media critics and, where available, by mental health practitioners within professional publications that also review films.^8^ As we see in the conflicted reception of *Jennifer*, dismissal or purposeful misrepresentation of the psychotherapeutic practice are enmeshed with broader conservative views on the family, gender and children. Perhaps these reviewers regarded the intersecting practices of progressive child psychology and socially-engaged filmmaking – demonstrated in the ongoing ‘child psychology cycle’ – as a symptom of a broader feminisation of culture. This has an interesting parallel in the documentary strand of this cycle where the Central Office of Information instinctively looked to female directors to document child psychotherapy practice which was, at this time, the only area of the mental health field in which women were able (or perhaps permitted) to be as prominent as men.

In terms of ethics, I have argued that fiction film might offer a possibility to reduce (rather than necessarily resolve) some of the ethical issues around documenting real patients and obtaining informed consent and permission to screen recorded material beyond its medical setting. Other contemporaneous films – such as the Ealing drama *Mandy* (1952), which focuses on the now controversial approach to deaf education of ‘oralism’ and featured a hearing child actor portraying the titular character – were filmed on location (in this case, at the Royal Residential Schools for the Deaf, Manchester) and cast and credited people with current or former lived experiences therein (present pupils and residents) (Cartwright, 2008). This takes such scenes much closer to the genre of docudrama, raising more pronounced questions of informed consent (who agreed the pupils involvement in *Mandy*? The School? Parents?) than *Jennifer* which employs child actors throughout. However, the script for *Jennifer* draws upon real people and real case history material. Furthermore, if as above, we deduce that Jennifer’s Worlds were based (at least in part) on Mary Smith’s or composites of other children, then the film is opening up their patient records to public scrutiny, in a way that they will likely have neither imagined nor consented to when they created and discussed them with Lowenfeld. The patient record debate (Sadowsky and Smith, 2024) is one that the Science Museum considered when they recreated some of the Worlds published in Lowenfeld’s own writing for public display on the Wellcome galleries.

Conversely, it is these very materials that allow the filmmakers (and resultantly audiences) to understand and empathise with the protagonist, and to question doctor-patient hierarchies and their relationships to broader social institutions of power (including the family). In *Jennifer*, the Doctor ends her discussion with a colleague about the origins of Jennifer’s emotional problems with the quip, ‘It’s the parents who should be in the clinics, not the children’. In this seemingly flippant comment, the Doctor hints at a radical aspect to Lowenfeld’s thought, in its identification of toxic family dynamics not only as the likely source of children’s ‘maladjustment’, but also in its insistence that children should not be made to adjust to those environments but be offered alterative ones in which they can be free to discover, develop and express themselves. Although outside the scope of this article, we might see some interesting connections here between Lowenfeld’s ideas, and the anti-psychiatric view of the family as potentially pathological (Wall, 2017: 195–196).

**Figure 5.**
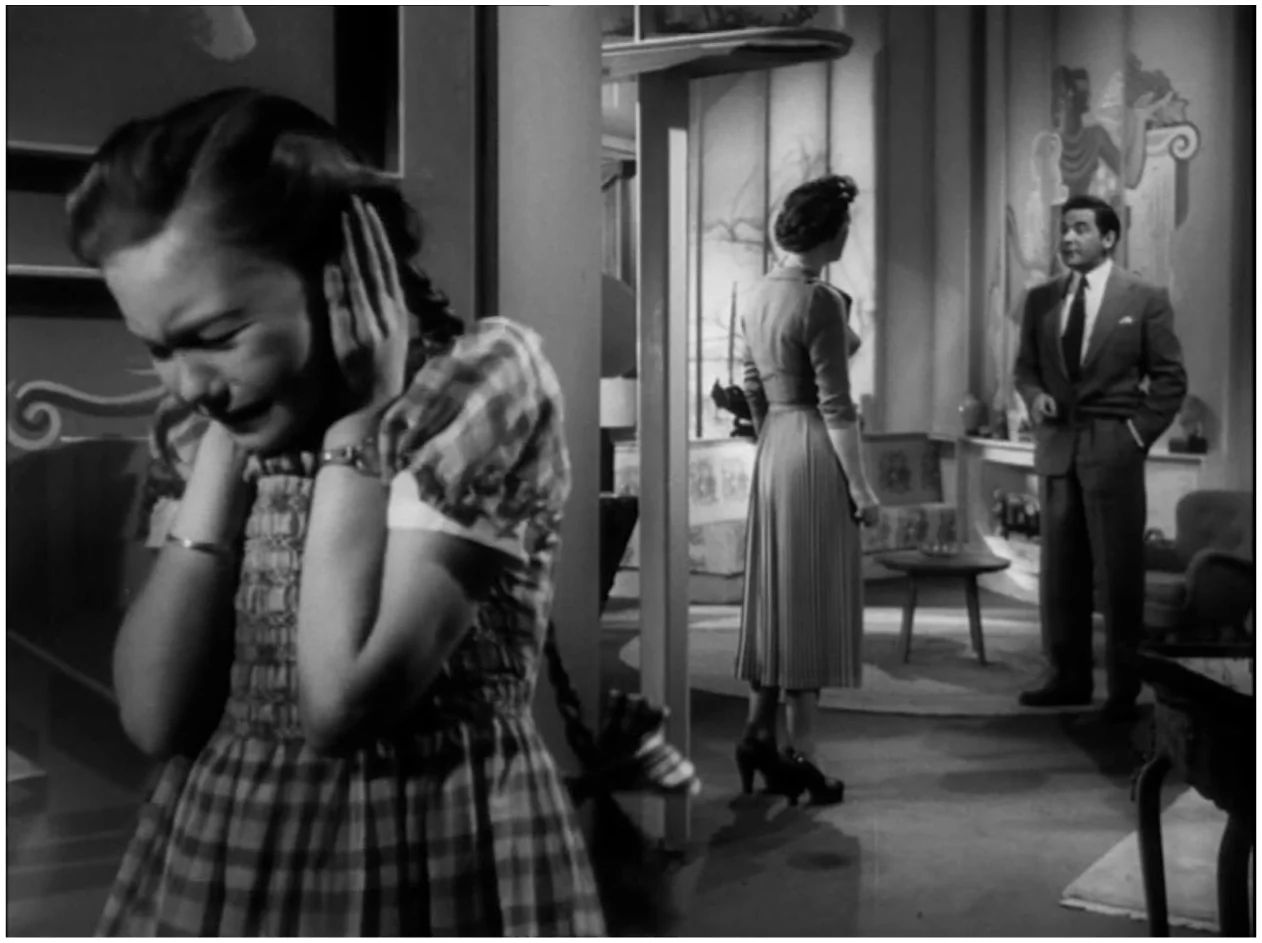
Expressive framing is used to generate empathy with Jennifer. Source: Image courtesy of StudioCanal.

In the short sequences set in the ‘ICP’, Jennifer experiences and co-creates environments that allow her to escape being ‘split in two’ (as the Doctor provokes) by family conflict and contradictory messaging within the parental home. Prior to her referral there, *Jennifer* uses subjective techniques (such as the aforementioned dream sequence), and expressive framing such that we experience Jennifer’s inner turmoil that stems, as the Doctor subsequently explains, from being ‘fought over, split in two, never left alone, never allowed to be herself. Something for the parents to argue about’ (see Figure 5). A comparable effect is achieved through *Mandy*’s sound design, selectively using a technique the director Alexander Mackendrick defined as ‘subjective non-sound’ (Garrido, 2003), to re-create the deaf child’s experience of silently navigating a fractious home environment and her initially frustrating experience of deaf education. Through such artistry, the spectator is invited to experience these ‘real’ therapeutic spaces through emotional engagement with the film’s eponymous child protagonists. This isn’t to say that documentaries or other archival films don’t seek to offer emotional identification with their subjects, but that they tend to rely more on exposition and explanation than experiential techniques and empathic alignment. One medium is not more valuable than the other, but embracing the potential of documentary *and* fiction films might aid historians in building a composite picture of past therapeutic cultures that might be both more accessible and inclusive.

## Conclusion

This article has demonstrated that discoveries within largely forgotten genre films can help to resituate real scientific spaces, practices and objects within their historical contexts, whilst offering insight into the contestations and controversies they provoked across a range of reception contexts.^9^ We can consider the World-building scenes in *Jennifer* as allying the twin apparatuses of the World Technique and the camera to ‘give a child power to express [her] ideas and feelings’ (Lowenfeld, 1950) outside repressive adult systems of thought, including the traditional family unit, psychoanalysis, (male) critics’ taste distinctions, and so forth. Since *No Place for Jennifer*’s release, the apparatus of the WT has been invoked as a visual shorthand for the psychologically disturbed child across a range of psychological thrillers, horror and science fiction films, from the schlocky 1970 sci-fi drama *The Mind of Mister Soames* (1970) to Jordan Peele’s psychological horror film *Us* (2019). The visual shorthand of the WT in these genre films has an equivalence with the Hollywood horror leitmotif of the ‘abandoned asylums of the Kirkbride Plan’ discussed by Pérez-Fernández and López-Muñoz (2019). In the intertextual evocation of the WT in the psychological set up of recent film like *Us*, for example, we can appreciate both the lasting global legacy of Lowenfeld’s technique and its dilution and disconnection from her original ideas and intentions. This makes *Jennifer*’s meticulous care, precision and authenticity in detailing both place and practice as all the more important as a historical record of the ICP and WT.

In addressing the limitations of the term ‘documentary’, Baron argues for the term ‘archival document’, given the greater slippages of the term ‘document’ in its simultaneous evocation of a physical object, a historical event and subjective intent (to document, access or reuse for a particular purpose). The ‘archive’ part of her term draws, perhaps unsurprisingly, on Foucault’s (2006) definition of ‘the archive’, as ‘the first law of what can be said’ (p. 28) about the past, or, quoting Baron, ‘as a particular structure of power in which particular kinds of documents are kept in a particular order, thereby delimiting the possibilities of what may be said about the documents and, indeed, of knowledge itself’ (pp. 2–3). The field of the history of psychiatry (and allied disciplines) has for some time appreciated that documentary film has played important roles in the development, documentation and dissemination of clinical ideas, so this article is nudging this logic just a little further in arguing for fiction films to be evaluated for their potential value as a form of documentary evidence. Whilst dramatisations like *Jennifer* or *Mandy* open up their own epistemic and ethical issues (the limitations of genre filmmaking, the casting of child actors and/or non-actors, the repurposing of patient records, etc.), they also offer a frame to view alternate and excluded histories that can expand not only ‘what can be said’ about the past but also challenge the language in which it can be said.

## Notes

1. I was fortunate to present an early version of this paper at the ‘Patients & Medical Staff: Interrogating Power Dynamics in 20th Century Psychiatry and Medicine through Audiovisual Archives’ conference (Lausanne, 27–28 March 2025) which was dedicated to exploring this issue of the ‘medical gaze’ and/in archive films. Thanks to Mireille Berton and the whole ‘Cinema and (neuro)psychiatry in Switzerland: around the Waldau collections’ team.

2. The archives consulted for this article were: BFI National Archives; BBC Written Archives; the ‘Margaret Lowenfeld papers at The Wellcome Library; StudioCanal publicity files.

3. A 1955 ICP brochure explained, ‘The Centre believes that for this work to be done with success not only is a technically trained staff essential but also that the building in which the work is done should be properly adapted and equipped’ (Lowenfeld papers, PP/LOW, Wellcome Collection, Euston Road, London).

4. Ongoing anxieties across the wartime and post-war period by politicians, sociologists and the media about the content of films seen by children and their behaviour within cinema clubs encouraged the government to set up a Departmental Committee on Children and Cinema which, in its 1950 report, concluded that cinemagoing could be good for children but stressed an ‘urgent need to produce quality film entrainment for’ them. This resulted in the founding of the Children’s Film Foundation in 1951 charged to make ‘healthy films’ for children’s cinema clubs and matinees (Roberts, 2017: 202).

5. Stage direction in the script suggest that her third World had ‘become what no doubt the town-planning people would have liked it to be’.

6. In Thomson’s later training film for nurses, *Understanding Aggression* (1960), she shows nurses watching *Children Learning by Experience* (1946) as part of their training.

7. Correspondingly, most reporting on Lowenfeld’s work in the ICP in British newspapers is ‘relegated’ to the Women’s section, suggesting that children’s mental health is the concern of women only.

8. In the UK, for example, the National Association for Mental Health (later MIND) reviewed relevant films in its publication, Mental Health (also later MIND), from the mid-1940s onwards.

9. Another strong demonstration of this point is 1955 British sci-fi crime film *The Brain Machine* which offers insight into the practice and perception of the EEG as a female expertise in the 1950s, but also shows how the film (and its title) demonstrate and contribute to the dominant media and public understanding of the EEG at this time as a device used to detect criminal psychopaths (Snelson et al., 2024b).
